# Regulating the effects of GPR21, a novel target for type 2 diabetes

**DOI:** 10.1038/srep27002

**Published:** 2016-05-31

**Authors:** Siobhán Leonard, Gemma K. Kinsella, Elisa Benetti, John B. C. Findlay

**Affiliations:** 1 Department of Biology, National University of Ireland Maynooth, Maynooth, Co. Kildare, Ireland; 2 Department of Drug Science and Technology, University of Turin, Turin, Italy

## Abstract

Type 2 diabetes is a chronic metabolic disorder primarily caused by insulin resistance to which obesity is a major contributor. Expression levels of an orphan G protein-coupled receptor (GPCR), GPR21, demonstrated a trend towards a significant increase in the epididymal fat pads of wild type high fat high sugar (HFHS)-fed mice. To gain further insight into the potential role this novel target may play in the development of obesity-associated type 2 diabetes, the signalling capabilities of the receptor were investigated. Overexpression studies in HEK293T cells revealed GPR21 to be a constitutively active receptor, which couples to Gα_q_ type G proteins leading to the activation of mitogen activated protein kinases (MAPKs). Overexpression of GPR21 *in vitro* also markedly attenuated insulin signalling. Interestingly, the effect of GPR21 on the MAPKs and insulin signalling was reduced in the presence of serum, inferring the possibility of a native inhibitory ligand. Homology modelling and ligand docking studies led to the identification of a novel compound that inhibited GPR21 activity. Its effects offer potential as an anti-diabetic pharmacological strategy as it was found to counteract the influence of GPR21 on the insulin signalling pathway.

Type 2 diabetes is primarily caused by a systemic insulin resistant state provoked by increasing viseral adipose tissue that triggers chronic, low-grade inflammation, which negatively impacts on the insulin signalling pathway^1^,^2^. The rising incidence of type 2 diabetes, along with the limitations of current treatment regimes, urge the need for innovative, effective strategies to prevent the development and progression of this disease. G protein-coupled receptors (GPCRs), the largest protein superfamily in the genome, represent a rich source of drug targets as they readily convey external signals to the internal environment of the cell: approximately 30–40% of marketed drugs target these versatile receptors^3^.

Analysis of the G protein to which a GPCR couples to amplify signal potential is key to understanding the activity and downstream consequences of receptor activation, as well as providing a means to assess the functional impact of any ligands postulated to bind to the receptor. Selective GPCR coupling to Gα_q_ subtype G proteins leads to the activation of phospholipase C (PLC)^4^, which cleaves phosphatidylinositol 4,5-bisphosphate (PIP_2_) into the secondary messengers, diacylglycerol (DAG) and inositol 1,4,5-trisphosphate (IP_3_). The membrane bound DAG activates protein kinase C (PKC), whereas, the soluble IP_3_ binds to its receptor in the endoplasmic reticulum triggering the release of Ca^2+^ 5. Downstream of this, a wide range of intracellular pathways can be activated, including the mitogen activated protein kinase (MAPK) cascade^6^. The MAPK family comprises three members; extracellular-signal-regulated protein kinase (Erk), p38, and c-Jun NH_2_-terminal kinase (JNK), which play crucial roles in cell proliferation, oncogenesis, differentiation, inflammation, stress responses and cell migration^7^,^8^. Notably, JNK is recognised as a major contributor to insulin resistance as it induces the phosphorylation of insulin receptor substrate 1 (IRS1) at Ser^307^. This prevents insulin-stimulated tyrosine phosphorylation of the protein, thus attenuating the insulin signalling pathway^9^.

We have observed an increase in the expression levels of an orphan GPCR, GPR21, in the adipose tissue of high fat high sugar (HFHS)-fed mice. Although this increase did not reach a statistically significant level, GPR21 may represent a novel means by which the type 2 diabetic phenotype could be targeted as this GPCR has been suggested to couple with the Gα_q_ subtype G proteins, Gα_q_^10^ and Gα_15/16_^11^. Advances in homology modelling and ligand docking studies have greatly facilitated the development of targeted therapies towards orphan GPCRs^12^. As the structure of GPR21 remains unknown, these techniques were employed to identify potential small molecules capable of binding to and regulating the effects of this receptor.

This work provides an analysis of GPR21-induced signal transduction, yielding an insight into the mechanisms by which this receptor could exert an effect in the type 2 diabetic phenotype and thus may represent an opportunity for a new therapeutic strategy. The observed constitutive activity of GPR21, which promotes MAPK activation and negatively impacts on the insulin signalling pathway, may be regulated by a native ligand present in serum. Furthermore, a novel compound designed to bind to GPR21 has been found to protect against the observed effects of the receptor on the insulin signalling pathway.

## Results

### GPR21 is a constitutively active receptor signalling through Gα_15/16_

**Figure 1:**
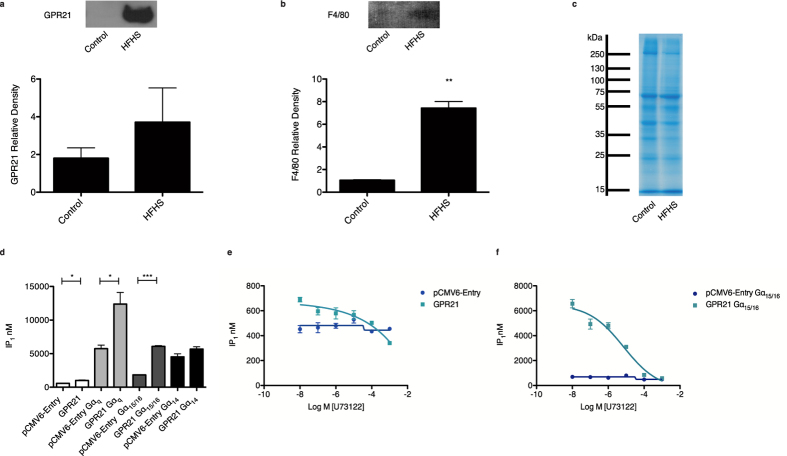
Analysis of GPR21 expression and activity. Epididymal fat pads of control and HFHS-fed C57BL/6J mice were lysed, proteins separated by SDS-PAGE and expression of (**a**) GPR21 and (**b**) the macrophage marker, F4/80, evaluated by Western blotting. Representative Western blots are shown along with relative densities, determined using Image J software, presented as mean ± SEM, n = 9. Using the unpaired Student’s *t*-test a significant increase in F4/80 expression was observed at p < 0.01**. (**c**) Representative Coomassie stained gel used to confirm equal loading of protein extracted from murine epididymal fat pads. (**d**) A FRET-based IP-one assay was employed to assess the activity of GPR21 in transiently transfected HEK293T cells when coupled with the Gα_q_ G protein subtypes, Gα_q_, Gα_15/16_ or Gα_14_. HEK293T cells were also transfected with the empty vector, pCMV6-Entry. Data presented as mean ± SEM are representative of three independent experiments performed in triplicate each time. One-way ANOVA with a post-hoc Tukey test denotes a significant increase in GPR21-induced IP_1_ production at p < 0.05* and p < 0.001***. As Gα_q_ stimulation induces PLC activation to trigger the inositol cascade, the effect of the PLC inhibitor, U73122, on IP_1_ production was also assessed in HEK293T cells transiently transfected with (**e**) pCMV6-Entry or GPR21 and (**f**) pCMV6-Entry or GPR21 coupled with Gα_15/16_. GraphPad Prism^®^ 5 software was used to generate a non-linear sigmoidal dose response curve in response to U73122. Data presented as mean ± SEM are representative of two independent experiments performed in triplicate each time.

Analysis of the epididymal fat pads of wild type C57BL/6J mice, a meaningful indicator of obesity-related diabetes, revealed an increase in GPR21 expression, which trended towards significance, in HFHS-fed mice (Fig. 1a), with a concurrent elevation in the macrophage marker F4/80 (Fig. 1b). Following on from this, an investigation into the G protein to which GPR21 is believed to couple was undertaken to give further insight into the functional consequence of a potential increase in expression. Analysis of inositol-1-phosphate (IP_1_) production was used as a surrogate of the transient secondary messenger IP_3_ to monitor activation of the Gα_q_ pathway^13^. The orphan receptor, GPR21, demonstrated constitutive activity when overexpressed in HEK293T cells as indicated by an increase in endogenous IP_1_ in the absence of a ligand (Fig. 1d). To determine the spectrum of Gα_q_ proteins GPR21 is capable of signalling through, HEK293T cells were co-transfected with cDNAs of the α-subunits of the G_q_ family, Gα_q_, Gα_15/16_ and Gα_14_. When expressed with the empty vector, pCMV6-Entry, all Gα_q_ subtypes led to an increase in basal IP_1_ levels. When coupled with GPR21, an increase in IP_1_ production was observed in HEK293T cells overexpressing Gα_q_. This indication of GPR21 constitutive activity further increased when the receptor was coupled with Gα_15/16_. Gα_14_ did not appear to influence GPR21-induced production of IP_1_. The PLC inhibitor, U73122, led to a dose-dependent decrease in IP_1_ production in HEK293T cells overexpressing both GPR21 alone (Fig. 1e) and coupled with Gα_15/16_ (Fig. 1f), reinforcing the pathway involved.

### Overexpression of GPR21 leads to activation of the MAPKs and negatively impacts on the insulin signalling pathway

**Figure 2:**
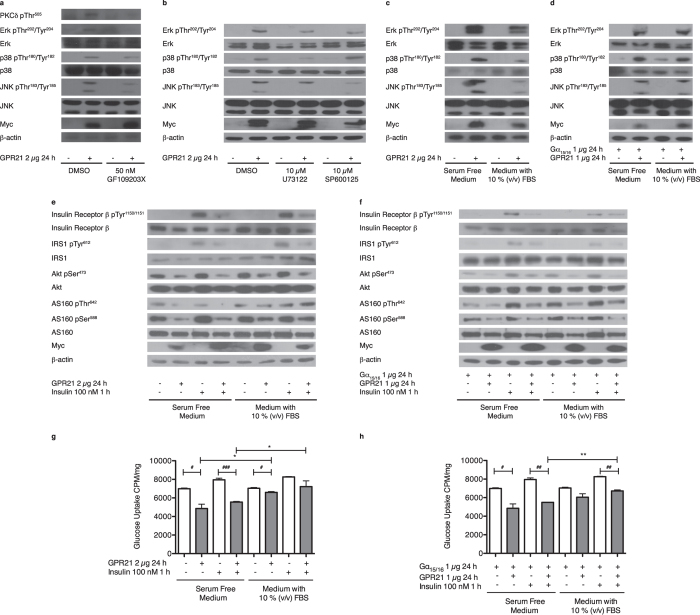
Analysis of the effect of GPR21 on the MAPKs, insulin signalling and glucose uptake. (**a**) HEK293T cells transiently transfected with pCMV6-Entry or GPR21 were incubated with 50 nM GF109203X or an equal volume of the vehicle control, DMSO, for 16 h in serum free medium. (**b**) HEK293T cells transiently transfected with pCMV6-Entry or GPR21 were incubated with 10 μM U73122, 10 μM SP600125 or an equal volume of DMSO in serum free medium for 24 h. (**c**) HEK293T cells transiently transfected with pCMV6-Entry or GPR21 and (**d**) pCMV6-Entry or GPR21 coupled with Gα_15/16_, were incubated for 24 h in the presence or absence of 10% (v/v) FBS. Cells were lysed and subjected to SDS-PAGE followed by immunoblotting with antibodies against phospho-PKCδ Thr^505^, phospho-Erk Thr^202^/Tyr^204^, Erk, phospho-p38 Thr^180^/Tyr^182^, p38, phospho-JNK Thr^183^/Tyr^185^, JNK, myc and β-actin. Western blots are representative of two separate experiments. (**e**) HEK293T cells transiently transfected with pCMV6-Entry or GPR21 and (**f**) pCMV6-Entry or GPR21 coupled with Gα_15/16_, were incubated for 24 h in the presence or absence of 10% (v/v) FBS, then stimulated with 100 nM insulin for 1 h. Cells were lysed and subjected to SDS-PAGE followed by immunoblotting with antibodies against phospho-Insulin Receptor β Tyr^1150/1151^, Insulin Receptor β, phospho-IRS1 Tyr^612^, IRS1, phospho-Akt Ser^473^, Akt, phospho-AS160 Thr^642^, phospho-AS160 Ser^588^, AS160, myc and β-actin. Western blots are representative of three separate experiments. Alternatively, the consequential effect on glucose uptake was established by measuring cellular levels of [^3^H]-2-deoxyglucose in cells transiently transfected with (**g**) pCMV6-Entry or GPR21 and (**h**) pCMV6-Entry or GPR21 coupled with Gα_15/16_. Data presented as mean ± SEM are representative of two independent experiments performed in triplicate. One-way ANOVA with a post-hoc Tukey test conveys a significant increase in glucose uptake at p < 0.05* and p < 0.01**. A significant decrease in glucose uptake is denoted at p < 0.05^**#**^, p < 0.01^**##**^ and p < 0.001^**###**^.

Downstream of GPR21-mediated PLC activation meaningful effects on PKCδ and the MAPKs were observed. Overexpression of GPR21 led to a considerable increase in the phosphorylation of PKCδ, Erk, p38 and JNK, an effect that could be attenuated with 50 nM of the PKC inhibitor GF109203X (Fig. 2a). Incubation with 10 μM U73122 also reduced the impact of GPR21 on the MAPKs, the effect on JNK being similar to that of the JNK inhibitor, SP600125 (Fig. 2b). The influence of GPR21 overexpression on the MAPKs was markedly reduced in the presence of serum (Fig. 2c), suggesting the existence of an inhibitory ligand for this constitutively active receptor. When coupled with Gα_15/16_ (Fig. 2d), GPR21 overexpression again led to an increase in the phosphorylation of Erk, p38 and JNK. However, in the presence of serum, a minimal reduction in phosphorylation was seen.

As overexpression of GPR21 caused a marked increase in JNK phosphorylation, the influence of the receptor on the insulin signalling pathway was assessed. HEK293T cells overexpressing GPR21 did not respond to insulin-induced stimulation of its receptor, a result that was somewhat reduced in the presence of serum (Fig. 2e). Further down the pathway, GPR21 overexpression impeded the phosphorylation of IRS1, Akt and AS160 in the presence and absence of insulin. This outcome was again reduced when cells were incubated with medium containing serum. A related result was observed when GPR21 was coupled with Gα_15/16_ (Fig. 2f), although the impact of serum was modest. As a consequence of an attenuated insulin signalling pathway, GPR21 also caused a significant decrease in glucose uptake, which partially recovered in the presence of serum (Fig. 2g). When co-expressed with Gα_15/16_ a similar trend was observed (Fig. 2h). The influence of GPR21 on the insulin signalling pathway is not limited to HEK293T cells, as this effect can also be seen in CHO cells overexpressing the receptor (see Supplementary Fig. S1).

### A novel compound protects against the effects of GPR21

**Figure 3:**
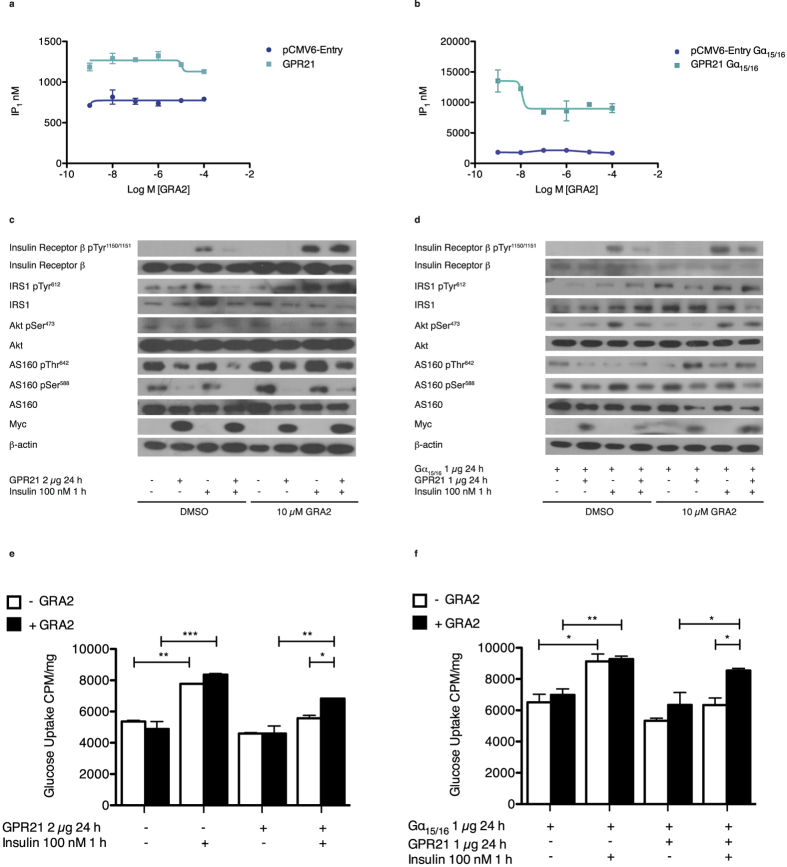
Analysis of the effect of a novel compound in HEK293T cells overexpressing GPR21. The direct effect of GRA2 on IP_1_ production was assessed using a functional FRET-based IP-one assay with HEK293T cells transiently transfected with (**a**) pCMV6-Entry or GPR21 and (**b**) pCMV6-Entry or GPR21 coupled with Gα_15/16_. To analyse the downstream effects of GRA2 on regulating the impact of GPR21 on the insulin signalling pathway, HEK293T cells overexpressing (**c**) pCMV6-Entry or GPR21 and (**d**) pCMV6-Entry or GPR21 coupled with Gα_15/16_ were incubated with 10 μM GRA2 or an equal volume of the vehicle control in serum free medium for 24 h followed by a 1 h stimulation with 100 nM insulin. Cells were lysed and subjected to SDS-PAGE followed by immunoblotting with antibodies against phospho-Insulin Receptor β Tyr^1150/1151^, Insulin Receptor β, phospho-IRS1 Tyr^612^, IRS1, phospho-Akt Ser^473^, Akt, phospho-AS160 Thr^642^, phospho-AS160 Ser^588^, AS160, myc and β-actin. Western blots are representative of two separate experiments. To establish the direct impact on glucose uptake, incorporation of [^3^H]-2-deoxyglucose was evaluated via scintillation counting of solubilised cells overexpressing (**e**) pCMV6-Entry or GPR21 and (**f**) pCMV6-Entry or GPR21 coupled with Gα_15/16_. Data presented as mean ± SEM are representative of two independent experiments performed in triplicate. A significant increase in glucose uptake was observed at p < 0.05*, p < 0.01** and p < 0.001***.

Virtual screening of an in house model of GPR21 identified 11 compounds with the potential to bind to GPR21. One compound, GRA2, appeared to act as an inverse agonist of the orphan receptor. This novel compound decreased IP_1_ production in cells overexpressing GPR21 (Fig. 3a), a finding more pronounced when the receptor was coupled with Gα_15/16_ (Fig. 3b). In the presence of GRA2, GPR21 had no effect on insulin-stimulated phosphorylation of its receptor, whereas GPR21 overexpression diminished the effect of insulin in cells incubated with the DMSO control (Fig. 3c). GRA2 counteracted the GPR21 effect on IRS1, and the phosphorylation of Akt and of AS160 at two residues critical to facilitate the translocation of the glucose transporter, GLUT4, also improved, although, to a lesser extent. In addition to this, HEK293T cells transfected with pCMV6-Entry demonstrated a slight response to GRA2, in that phosphorylation of the components of the insulin signalling cascade increased somewhat, potentially due to a low level of endogenous receptor. A similar response was seen in cells co-transfected with GPR21 and Gα_15/16_ (Fig. 3d). Treatment of HEK293T cells with 10 μM GRA2 for 24 h did not significantly alter glucose uptake in cells transfected with the empty vector. However, insulin-induced glucose uptake improved in cells overexpressing GPR21 when compared to the vehicle control, DMSO (Fig. 3e). A similar result was observed when GPR21 was coupled with Gα_15/16_ (Fig. 3f).

## Discussion

Overexpression of GPR21 *in vitro* has assisted in elucidating the underlying mechanisms by which this receptor may contribute to the development of insulin resistance and type 2 diabetes.

Analysis of IP_1_ production, a stable downstream metabolite of IP_3_ that accumulates upon Gα_q_ receptor activation, suggested that GPR21, overexpressed in HEK293T cells, could couple to and activate Gα_q_ in the absence of a ligand, a hallmark of constitutive activity. Co-expression with the specific members of the Gα_q_ family revealed a functional interaction between GPR21 and Gα_15/16_, thereby facilitating PLC signalling. The addition of the PLC inhibitor, U73122, inhibited GPR21-induced production of IP_1_, confirming the selectivity of GPR21 on this signalling cascade. Furthermore, GPR21 was found to increase the phosphorylation of PKCδ, leading to the activation of the MAPKs, Erk, p38 and JNK, an effect that was also found to be dependent on PLC signalling. In the presence of medium containing 10% (v/v) FBS, the effect of GPR21 on MAPK activation was less significant, particularly in cells overexpressing the receptor alone, indicating the possibility of an endogenous regulatory ligand for GPR21 in serum. The inhibitory influence of this potential ligand on GPR21 activity was also seen on the insulin signalling pathway and associated glucose uptake. The action of the ligand may be to inhibit the GPR21-induced increase in JNK activity. The possibility of an endogenous ligand suggests that the proposed effect of GPR21 may be tightly regulated under normal physiological conditions and may only become deleterious in cases where this signalling pathway is altered.

In addition to these observations, GPR21 knockout studies have generated data that implicate the receptor in the development of the type 2 diabetic phenotype^14^,^15^. Mice lacking this GPCR, fed on a high fat diet (HFD), demonstrate increased insulin sensitivity, improved glucose tolerance and a reduction in pro-inflammatory markers, when compared to wild type littermates. Osborn and colleagues^14^ proposed GPR21 to have a role in co-ordinating macrophage pro-inflammatory activity, an effect which could be attributed to the effect of the receptor on JNK activation, as increased expression of this MAPK has been reported to promote HFD-induced accumulation of macrophages in adipose tissue^16^.

Given the influence of GPR21 on insulin signalling, a molecule that blocks its constitutive activity in a similar manner to the prospective native ligand could be a novel and powerful pharmacological strategy for the treatment of type 2 diabetes. Constitutively active orphan GPCRs, such as GPR21 provide a direct route to drug discovery as their functionality can be understood without the need to identify endogenous ligands^17^. A novel compound, GRA2, designed through *in silico* screening to bind to GPR21, demonstrated potential as an inverse agonist of the orphan receptor as it reduced the accumulation of IP_1_ in HEK293T cells overexpressing the receptor alone and more evidently with Gα_15/16_. As a consequence of reduced receptor activity, the signalling capabilities of insulin improved in cells overexpressing GPR21 with and without Gα_15/16_, resulting in a meaningful restoration of insulin-induced glucose uptake. Cells transfected with the empty vector also displayed modestly enhanced phosphorylation of the proteins involved in the insulin signalling cascade in response to GRA2. This may be as a consequence of the low level of endogenous GPR21 expressed in HEK293T cells. Structure-activity relationship studies based on this compound should yield higher affinity agents capable of exerting a more powerful effect through GPR21.

**Figure 4:**
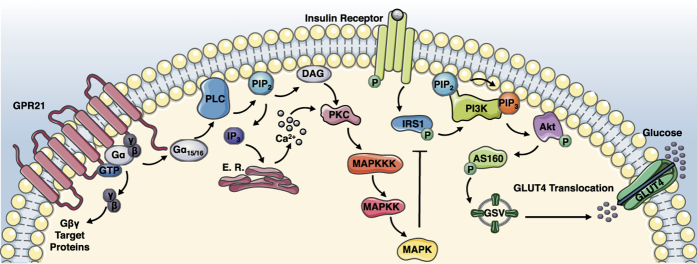
Proposed role for GPR21 in the development of type 2 diabetes. The constitutively active GPR21 recruits and activates Gα_15/16_, which facilitates the hydrolysis of PIP_2_ into DAG and IP_3_ through the action of PLC. Both DAG and IP_3_ activate PKC, which signals to and activates the MAPK cascade. The MAPK JNK plays a crucial role in the development of insulin resistance as it promotes the phosphorylation of a key component of the insulin signalling cascade, IRS1, at Ser^307^, thereby preventing insulin-induced tyrosine phosphorylation of this protein. GPR21-induced activation of the MAPKs may contribute to the development of type 2 diabetes. Due to the potential of a native inverse agonist in serum, GPR21 signal transduction is believed to be tightly regulated under normal physiological conditions. However in a state of obesity, elevated GPR21 activity may become deleterious, potentially promoting the genesis of insulin resistance.

This work demonstrates the potential role GPR21 may play in the development of type 2 diabetes (Fig. 4). Obesity-induced type 2 diabetes may be associated with the dysregulation of GPR21 through an increase in receptor expression, an increase in an endogenous agonist or a reduction in an inverse agonist, leading to the stimulation of the MAPKs, which contribute to the inhibitory influences of GPR21. Targeting GPR21 with an inverse agonist such as the one identified in this study could curtail some of the many facets contributing to the development of insulin resistance and type 2 diabetes.

## Methods

### Animals

Male C57BL/6J mice, aged 4 weeks (n = 18 provided by Charles River, Calco, Lecco, Italy) were housed in a temperature-controlled environment with a 12 h light/dark cycle. The animals were maintained on a pellet diet for 1 week, then randomly divided into two groups: normal diet (control, n = 9) and a high fat high sugar diet (HFHS, n = 9) for 16 weeks. The HFHS diet contained 45% kcal fat, 35% kcal carbohydrates and 20% kcal protein (D12451, ssniff Spezialdiäten GmbH, Germany). The procedures followed in this study were approved by the Animal Use and Care Committee of the University of Turin and subsequently by the ethics committee of the National University of Ireland, Maynooth (BSRESC-2014-008). Protocols were in accordance with the European Directive 2010/63/EU on the protection of animals used for scientific purposes.

### Cell culture, transfection and treatment

HEK293T cells were maintained in complete medium (high glucose Dulbecco’s Modified Eagle’s Medium (DMEM) containing 2 mM L-glutamine and 100 μg/ml penicillin-streptomycin) supplemented with 10% (v/v) foetal bovine serum (FBS) (Sigma), at 37 °C in a humidified 5% CO_2_ atmosphere. Cells were transfected with 2 μg human GPR21 containing a myc-tag incorporated into the C-terminus, or the empty vector, pCMV6-Entry (OriGene Technologies), with Lipofectamine^®^ 2000 (Invitrogen) according to the manufacturer’s protocols. When co-transfecting with cDNAs corresponding to the G protein α-subunits, Gα_q_, Gα_15/16_ and Gα_14_ (cDNA Resource Centre), 1 μg of each plasmid was used. Cells were incubated for 24 h at 37 °C to allow incorporation of the plasmid DNA. To determine the influence of serum on GPR21 activity, cells were incubated for a further 24 h with fresh complete medium ± 10% (v/v) FBS. Treatment with compounds (GF109203X, U73122 and SP600125, Sigma) was carried out in serum free complete medium. To assess the effect of GPR21 on insulin signalling, HEK293T cells were stimulated with 100 nM insulin (Sigma) in Kreb’s Ringer Buffer (KRB); 136 mM NaCl, 20 mM HEPES, 4.7 mM KCl, 1 mM MgSO_4_, 1 mM CaCl_2_, 4.05 mM Na_2_HPO_4_, 0.95 mM NaH_2_PO_4_, pH 7.4, containing 5 mM glucose for 1 h at 37 °C.

### Inositol Phosphate one (IP-one) Homogenous Time Resolved Fluorescence (HTRF) assay

Cellular IP_1_ levels were measured using an IP-one HTRF assay kit (Cisbio)^13^. Briefly, subconfluent cells were detached from the cell culture dish by trypsinisation and resuspended in the appropriate volume of the assay stimulation buffer (10 mM HEPES, 1 mM CaCl_2,_ 0.5 mM MgCl_2_, 4.2 mM KCl, 146 mM NaCl, 5.5 mM glucose, 50 mM LiCl, pH 7.4) warmed to 37 °C, to achieve a concentration of 6 × 10^6^ cells/ml. The cell suspension was added to a white half-volume 384 well plate (Greiner) along with the compound to be tested and incubated at 37 °C for 2 h. IP-one lysis buffer containing 5% IP-one-d_2_ conjugate was added to the appropriate wells, followed by 5% anti-IP-one cryptate Tb conjugate. Samples were incubated in the dark for 1 h at room temperature. The plate was read on BMG labtech plate reader with an excitation at 340 nm and emission at 615 nm and 665 nm respectively. The fluorescence resonance energy transfer (FRET) ratios (665 nm/615 nm) were converted to IP_1_ concentrations by interpolating values from an IP_1_ standard curve.

### Preparation of lysates and Western blot analyses

Frozen murine epididymal fat pads were homogenised in ice-cold lysis buffer (50 mM HEPES pH 7.5, 150 mM NaCl, 50 mM NaF, 1 mM EGTA, 1.5 mM MgCl_2_, 2 mM Na_3_VO_4_, 10% (v/v) glycerol,   mM Na_4_P_2_O_7_, 1 mM PMSF, 1X SigmaFAST protease inhibitor cocktail and 1% (v/v) Triton x-100) at a ratio of 1:5 (w:v) using a handheld T10 Basic Homogeniser (IKA). Cells were washed three times with ice-cold phosphate buffered saline (PBS) then resuspended in lysis buffer. Crude extracts were incubated for 1 h at 4 °C with constant agitation. Samples were centrifuged at 17,000 × g for 10 min at 4 °C, insoluble pellets were discarded, and protein concentration was determined using the Pierce protein assay (PN22660).

Lysates of equal protein concentration were separated by SDS-PAGE, transferred onto a PVDF membrane and blocked for 1 h in Tris-buffered saline (150 mM NaCl, 20 mM Tris-HCl, pH 7.6) with 0.1% (v/v) Tween-20, containing 5% (w/v) bovine serum albumin (BSA). Membranes were incubated with primary antibodies overnight at 4 °C followed by the secondary antibodies conjugated with horseradish peroxidase for 2 h at room temperature. Protein bands were visualised on x-ray film with enhanced chemiluminescence (Roche). Immunodetection was achieved using the following antibodies from Cell Signaling Technology; phospho-PKCδ Thr^505^ (9374), phospho-Erk Thr^202^/Tyr^204^ (4370), Erk (4695), phospho-p38 Thr^180^/Tyr^182^ (4511), p38 (9212), phospho-JNK Thr^183^/Tyr^185^ (4668), JNK (9258), phospho-Insulin Receptor β Tyr^1150/1151^ (3024), IRS1 (3407), phospho-Akt Ser^473^ (9271), Akt (9272), phospho-AS160 Thr^642^ (8881), phospho-AS160 Ser^588^ (8730), AS160 (2670) and myc (2276). Phospho-IRS1 Tyr^612^ (44-816G) was supplied by Life Technologies and Insulin Receptor β (ab69508), GPR21 (ab139654), F4/80 (ab74383) and β-actin (ab8226) were purchased from Abcam.

### 2-deoxyglucose uptake

Glucose uptake was measured according to Yun and colleagues^18^, with modifications. Transfected HEK293T cells were washed once with KRB warmed to 37 °C, then incubated with 1 μCi/ml [^3^H]-2-deoxyglucose (PerkinElmer, NET328A001MC), prepared in KRB, at 37 °C for 10 min. To terminate the assay, cells were washed 3 times with ice-cold KRB and lysed in 0.1% (w/v) SDS at 37 °C for 30 min. Cell lysates were diluted 1:4 in β-scintillation fluid (Beta-Plate Scint, PerkinElmer). Incorporation of [^3^H]-2-deoxyglucose was quantified with a Wallac 1450 Microbeta Liquid Scintillation Counter (PerkinElmer), expressing results in counts per minute (CPM). Total protein contents were determined by the Bicinchoninic Acid procedure^19^ to define results as CPM/mg.

### GPR21 homology modelling and ligand docking

An in-house GPR21 structural model was constructed using the Modeller software embedded in Biovia Discovery Studio^20^. A sequence alignment was constructed between the amino acid sequence of the GPCR templates (2RH1^21^, 4GBR^22^), with the hGPR21 (Swissprot accession code Q99679), and manually inspected to confirm the correct alignment of conserved residues^23^. Using the alignment, 1,000 different models were generated with a subsequent refinement protocol applied to the loop regions^24^,^25^. Disulfide bonds were formed between Cys^102^ and Cys^181^. The final model was selected using the Modeller objective score and a selection of protein assessment tools (http://services.mbi.ucla.edu/SAVES/)^26^,^27^.

Utilising this developed protein model, virtual screening processes were implemented to examine large compound databases (e.g. Specs, www.specs.net) *in silico* through molecular docking studies (OpenEye, Fred^28^,^29^,^30^). The molecules were standardised, and tautomers and stereoisomers were enumerated using Biovia Pipeline Pilot^20^. Conformers were generated using Omega^31^,^32^,^33^ from Openeye prior to docking using default parameters with Fred^29^ and scoring with the chemgauss3 scoring function. From the ranked list, 11 compounds were ordered for experimental validation.

### Data analysis

Data are presented as means ± standard error of the mean (SEM). The unpaired Student’s *t*-test was used to determine significance between groups. One-way analysis of variance (ANOVA) with a post-hoc Tukey test was used to determine significance among three or more groups. Significance was denoted as p < 0.05*, p < 0.01**, p < 0.001***.

## Additional Information

**How to cite this article**: Leonard, S. *et al.* Regulating the effects of GPR21, a novel target for type 2 diabetes. *Sci. Rep.* **6**, 27002; doi: 10.1038/srep27002 (2016).

## Supplementary Material

Supplementary Figure S1
