# Supplementary material for: Regulating the effects of GPR21, a novel target for type 2 diabetes
Source: Sci Rep. 2016 May 31;6:27002. doi: 10.1038/srep27002 (PMC4886680; doi:10.1038/srep27002)
Supplement: Supplementary Figure S1 [file srep27002-s1.pdf]

Regulating the effects of GPR21, a novel target for type 2 diabetes

Siobhán Leonard[1,*], Gemma K. Kinsella[1,♯], Elisa Benetti[2] and John B. C. Findlay[1]

[1]Department of Biology, National University of Ireland Maynooth, Maynooth, Co. Kildare, Ireland

[2]Department of Drug Science and Technology, University of Turin, Turin, Italy

[♯]Current address: School of Food Science and Environmental Health, College of Sciences and Health, Dublin Institute of Technology, Cathal Brugha Street, Dublin 1, Ireland

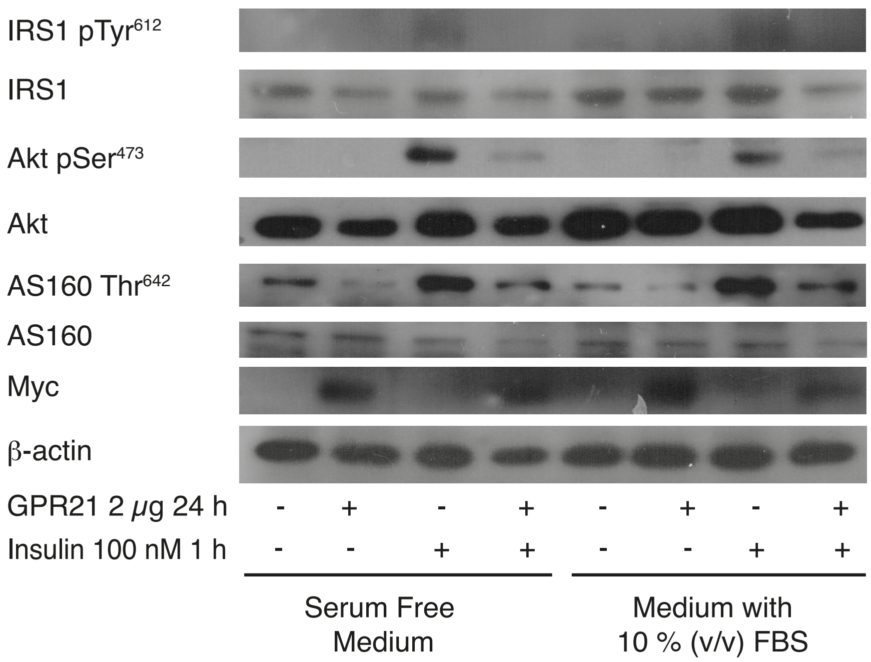

Supplementary Figure S1. Analysis of the effect of GPR21 on insulin signalling in CHO cells. CHO cells transiently transfected with pCMV6-Entry or GPR21 were incubated for 24 h in the presence or absence of 10 % (v/v) FBS, then stimulated with 100 nM insulin for 1 h. Cells were lysed and subjected to SDS-PAGE followed by immunoblotting with antibodies against phospho-IRS1 Tyr^{612}, IRS1, phospho-Akt Ser^{473}, Akt, phospho-AS160 Thr^{642}, AS160, myc and β-actin.
